# Reaching New Biocatalytic Reactivity Using Continuous Flow Reactors

**DOI:** 10.1002/chem.202103607

**Published:** 2022-01-10

**Authors:** Sebastian C. Cosgrove, Ashley P. Mattey

**Affiliations:** ^1^ Lennard-Jones Laboratory School of Chemical and Physical Sciences Keele University Keele Staffordshire ST5 5BG United Kingdom; ^2^ EnginZyme Tomtebodavägen 6 171 65 Solna Sweden

**Keywords:** Biocatalysis, Biocatalytic Cascades, Continuous Flow Biocatalysis, Immobilisation

## Abstract

The use of flow reactors in biocatalysis has increased significantly in recent years. Chemists have begun to design flow systems that even allow new biocatalytic reactions to take place. This concept article will focus on the design of flow systems that have allowed enzymes to go beyond their limits in batch. The case is made for moving towards fully continuous systems. With flow chemistry increasingly seen as an enabling technology for automated synthesis, and with advancements in AI‐assisted enzyme design, there is a real possibility to fully automate the development and implementation of a continuous biocatalytic processes. This will lead to significantly improved enzyme processes for synthesis.

## Graphical abstract

**Continuous flow** is a mainstay in the synthetic chemist's toolbox. It has also begun to see increasing application in biocatalysis. This manuscript discusses some of the challenges that continuous flow has contributed solutions to for the improvement of biocatalysis. It also poses how the future of continuous flow biocatalysis will change with new technologies such as automation, in‐line analysis and AI.

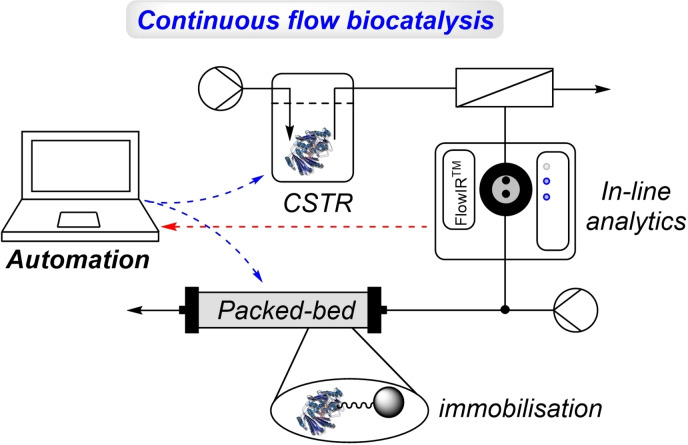

## Introduction

Enzymes are Nature's chemists, having evolved over millions of years to perform their specific functions with exquisite control under ambient conditions.^[1]^ Directed evolution has now also allowed access to non‐natural chemistry to expand the biocatalytic toolbox with examples ranging from C−N, C−C, and C−Si bond formation,^[2]^ to carbocyclic synthesis.^[3]^ It could be argued that with recent further advances such as de novo protein design,^[4]^ and expansion of the genetic code,^[5]^ there will be a point where even the most out of reach chemistries for enzymes (i. e., C_sp2_−C_sp2_ bond formation) could be engineered. Nevertheless, batch application of many of these enzymes leads to sub‐optimal performance.[^6^, ^7^] One frequently encountered issue is substrate/product inhibition. An area that can offer solutions to some of these issues is flow chemistry.

Flow involves running reactions with continuously moving phases in reactors as opposed to traditional, sequential batch operations with stationary media.^[8]^ There are multiple reactor types that can be used, but primarily tubular reactors are used, which can also contain solid supported reagents and catalysts in what are termed packed‐bed reactors. In general, flow allows for a greater control of the reaction conditions (e. g., temperature, reaction time) due to smaller rector sizes, can enable easier handling of hazardous reagents, and can facilitate efficient multi‐phase reactions. The rise of flow in synthesis is highlighted by the Food & Drug Administration recently producing quality assurance guidance for continuous manufacturing in relation to pharmaceutical production.^[9]^ Continuous processing has long been the method of choice in the petrochemical industry but has now matured into a key technique for synthesis, with the potential to improve around 50 % of all chemical reactions.[^6^, ^10^, ^11^, ^12^] The adoption of processes in continuous flow has a number of benefits over its batch counterpart with improved scalability and unique versatility in system design.^[8]^

There has been a steady increase in recent years in the number of reports of continuous biocatalytic reactions.[^10^, ^11^, ^12^, ^13^] What it has allowed chemists to do, as with traditional chemistry, is apply enzymes in more efficient ways. Enzymes can benefit from several improvements through transfer to a continuous process, such as improved volumetric productivity and easier reaction compartmentalisation.[^10^, ^11^] A key technology is enzyme immobilisation, which affords stability benefits to numerous enzymes and enables the use of packed‐bed reactors containing solid, heterogenous biocatalysts. Immobilisation has been reviewed comprehensively elsewhere, so will not be specifically discussed here.[^14^, ^15^]

Despite the benefits flow can bring, there must be a consideration as to how it will improve a process. For example, is an enzyme inhibited by a product so would benefit from continuous removal? Or is there a specific parameter that cannot be achieved in a batch reactor efficiently or safely (e. g., high pressure, poor scalability) that is more easily attained in a flow reactor? By addressing issues such as these, chemists have delivered innovative flow solutions for the improvement of multiple biocatalytic processes.

This article will focus specifically on how flow has enabled difficult (chemo)enzymatic transformations that would have been unproductive, and in some cases not possible, under normal batch conditions. As this is happening alongside the digital age, consideration of how artificial intelligence and automation have the potential to help further improve continuous biocatalytic reactions will also be considered.

### Multiphase Biocatalysis in Flow

One of the benefits of continuous processing is the greater control over multiple phases of reagents in the same reaction. This allows multiphasic reactions to be conducted with greater ease than in batch, for example with gas‐phase reagents. This section will discuss how different flow reactors have been used to enable multiphase reactions that would otherwise be difficult in batch.

#### Solid‐liquid biocatalysis in flow for co‐factor regeneration

Solid‐liquid phase reactions are perhaps the most used multiphasic reactions in biocatalysis, with immobilised enzymes an integral part of the biocatalytic toolbox.^[14]^ This has been the primary driver for transfer into flow reactors, with immobilised enzymes acting as heterogeneous catalysts that can be easily applied in packed‐bed reactors. The volume of work surrounding this area is too much to cover in this article alone. There are multiple recent, detailed articles that provide a comprehensive overview of the advances made in enzyme immobilisation for continuous flow, and we point towards these articles for further reading.[^10^, ^11^, ^12^, ^16^, ^17^] This section will therefore focus on recent examples that approach immobilisation from a non‐conventional aspect, specifically co‐factors.

A significant challenge associated with flow biocatalysis is the provision of cofactor. Nicotinamide (NAD(P)^+^/H) is prohibitively expensive for stoichiometric use, so is coupled to recycling systems to regenerate the required form to allow its use in catalytic quantities. Commonly used in oxidoreductase biocatalysis are glucose dehydrogenase (GDH) and formate dehydrogenase (FDH) for nicotinamide regeneration. These enzymes use co‐substrates glucose and formate to reduce the NAD(P)^+^ to the required NAD(P)H form. Even if a coupled process permits catalytic use, however, the continuous removal of the reaction media can still lead to high consumption of materials.

**Scheme 1:**
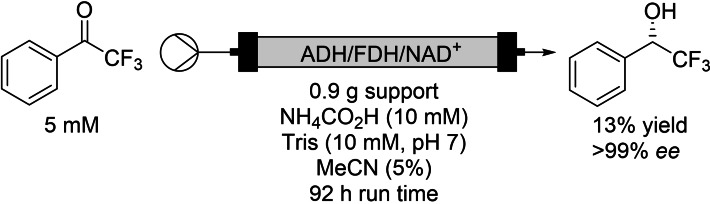
Continuous alcohol dehydrogenase with retained cofactors via ionic association.

Lopez‐Gallego and co‐workers recently reported a strategy to directly immobilise several important co‐factors for biocatalytic applications, namely NAD^+^, pyridoxal phosphate (PLP), which is important for aminotransferases, and flavin adenine dinucleotide (FAD), which is common in several classes of biocatalysts including oxidases.^[18]^ Exploiting the phosphorylated side‐chains of these molecules was key to their successful immobilisation, by using an ionic association with different ion exchange materials. The authors tested several resins and found an agarose support activated with polyethyleneimine was the most effective support for the three different co‐factors, with immobilisation yields of 18 μmol g_support_^−1^, 43 μmol g_support_^−1^ and 81 μmol g_support_^−1^, for NAD^+^, FAD and PLP, respectively. Of the three cofactors tested, PLP was the most stable with 99 % retention to the support after eight washes with buffer. Around 80 % of both NAD^+^ and FAD were eluted after eight wash cycles. Batch testing of the NAD^+^ with an alcohol dehydrogenase/formate dehydrogenase process for alcohol reduction showed much lower turnover frequency (TOF, calculated as μmol of product per μmol cofactor in one hour) with 0.11 min^−1^ for the soluble enzymes but only 0.064 for the immobilised. There was a four‐fold improvement with respect to total turnover numbers (TTN, mole of product per mole of NAD^+^), with an increase of 10 to 40. This specific process was transferred to a continuous reactor, and run for over 90 h with >90 % conversion to the *S*‐alcohol (Scheme 1). The reaction was run at a low substrate concentration (5 mM), but the isolated yield of 13 % (31 mg) was still poor considering 276 mL of effluent was collected. The productivity of the reactor therefore worked out to be 0.03 g_product_ g_support_^−1^, which is inefficient, but the retention of the cofactor meant this was able to be reused three more times without loss of activity. This would need to be significantly improved to be synthetically useful, but demonstrates an important principal in cofactor retention.

**Scheme 2:**
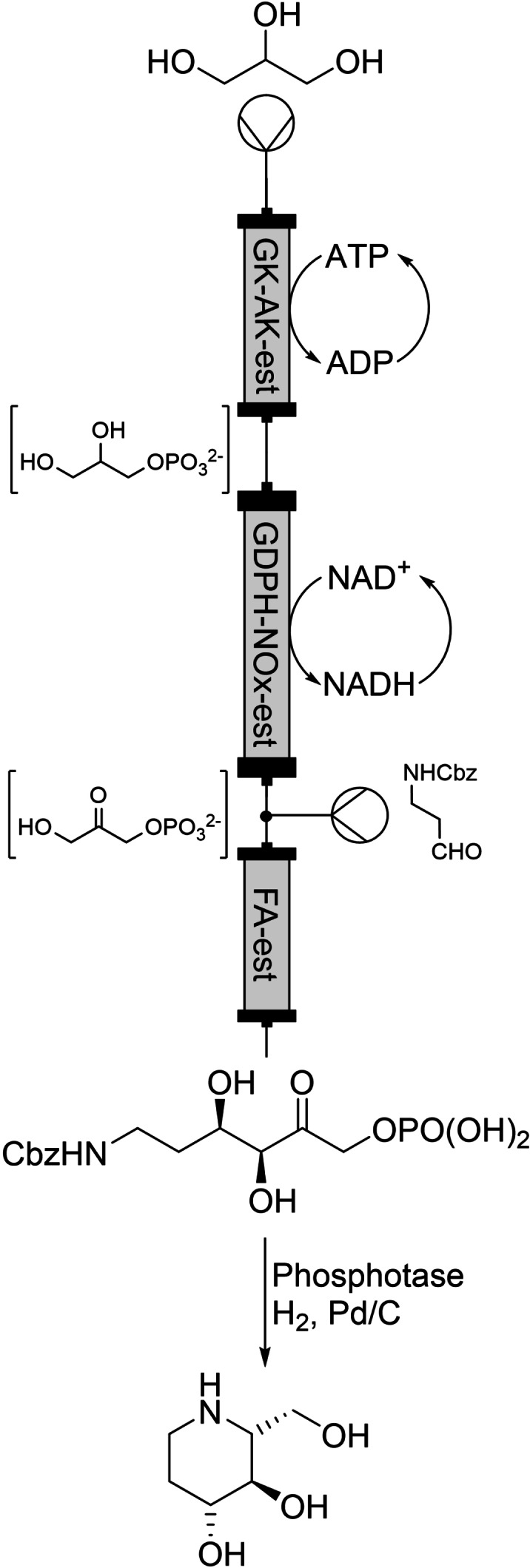
Immobilised enzyme modules containing recycling enzyme and tethered cofactor for d‐fagomine synthesis.

Another unique solution to this problem was presented by Scott and co‐workers, who genetically encoded fusion proteins to contain an active biocatalyst, the necessary cofactor, a recycling enzyme and an immobilisation conjugate.^[19]^ The fusion proteins were connected via short amino acid chains, with a modified cofactor also connected via a PEG linker. The immobilisation was achieved through covalent inhibition of a hydrolase enzyme with a site‐specific suicide inhibitor. In this instance it was a trifluoroketone that bound to a catalytic serine residue of the esterase from *Alicyclobacillus acidocaldarius* (est2_*Aa*_). The authors demonstrated the flexibility of the system through creating three consecutive modules which all contained immobilised fusion enzymes, and their respective co‐factors, that performed sequential reactions in the conversion of glycerol to d‐fagomine (Scheme 2).^[19]^ The three modules included a phosphotransfer reactor (glycerol kinase {GK}, acetate kinase {AK}, est2_*Aa*_, appended ATP), an oxidation reactor (glycerol‐3‐phosphate dehydrogenase {GPDH}, NADH oxidase {NOx}, est2_*Aa*_, appended NADH) and an aldol addition reaction (fructose aldolase {FA}, est2_*Aa*_). The final multistage reactor was able to produce a constant stream of >3 mM concentration of d‐fagomine for 440 minutes. The authors calculated that the total turnover numbers (TTN) for both ATP and NAD^+^ to be 16 848 and 10 389 respectively. They gave metrics specifically for the phosphotransfer reactor: it contained 36.9 mg of protein per column and yielded 2.6 g product per litre per hour. They calculated this to be an impressive productivity of 1399 nmol of product per nmol of enzyme. Overall, the aldolase process was calculated as having a space time yield of 28.6 mg L^−1^ h^−1^ mg_enz_^−1^, which was three times higher than a previously reported enzymatic flow synthesis of d‐fagomine, and 5.3 times more productive than the previous best reported batch enzymatic synthesis.

A key benefit of this system over previous studies that focussed on immobilised cofactors was the retention of activity compared to the non‐immobilised enzymes. The authors also compared their flow system to other biocatalytic non‐natural carbohydrate syntheses, demonstrating improvements in the STY versus the other methods. It could be argued that the necessity for enzyme engineering to produce the fusion modules renders this too specialist for some users. However, the cost and efficiency savings versus other methods could be great.

The retention of co‐factors on solid supports no doubt increases the efficiency of any bioprocess due to the reduced cost. What this needs to be attenuated against is the activity penalties that also may be encountered through immobilisation. Solutions such as those discussed here provide insight into how these applications could enhance different processes in future with further optimisation and research into scale.

#### Gas‐dependent biocatalysis in flow

Gas‐phase reactions provide significant challenges for synthetic chemists. Aside from the obvious safety issues that arise with using pressurised vessels, the solubility of gases in liquids is often limiting when considering gases as reagents. It is also hard to control exact amounts of gases in reactions. Flow can be used to deliver more precise control of gas.^[20]^ This has also allowed for several examples in the improvement of gas‐dependent enzymes in synthesis, which will be discussed below.

### Oxygen

The use of molecular oxygen as a sole terminal oxidant is a highly attractive proposition due to its abundance and low cost. Many oxygen‐dependent enzymes exist as such. They also often do not require the addition of expensive cofactors such as nicotinamide, further enhancing their economic and sustainability credentials.^[21]^ Experimentally, however, oxygen supply can lead to significant processing challenges due to the availability of molecular oxygen being limited by its poor solubility in aqueous media (ca. 270 μM).^[22]^ There are two approaches to improving oxygen availability: the first is increasing the rate of oxygen mass transfer to ensure that maximum solubility is maintained throughout a reaction, and the second is to increase the availability of soluble oxygen. Innovative reactor designs have provided solutions in both senses.

Some examples of reactors for improved mass transfer include a falling film reactor,^[23]^ which achieved maximum oxygen saturation of a reaction mixture in only 6 seconds, and an agitated cell reactor which was more scalable and also achieved a greater oxygen supply rate than batch reactors.[^24^, ^25^] The issue around improving mass transfer only, is that whilst a constant oxygen supply is maintained this never exceeds the maximum oxygen concentration in water under atmospheric pressure. Activity problems arise if the enzyme is kinetically limited, for example if the Michaelis constant for oxygen (*K*_MO_) is above 270 μM, which is the case for some oxidase enzymes. Woodley and co‐workers demonstrated this for glucose oxidase using a continuous tube‐in‐tube reactor (see below),^[26]^ calculating a *K*_MO_ value of 0.51 mM, around double the maximum aqueous concentration of oxygen and meaning that *V*_max_ is approximately four times higher than this. This implies that under ambient conditions, glucose oxidase is oxygen‐limited and therefore impaired.

**Figure 1:**
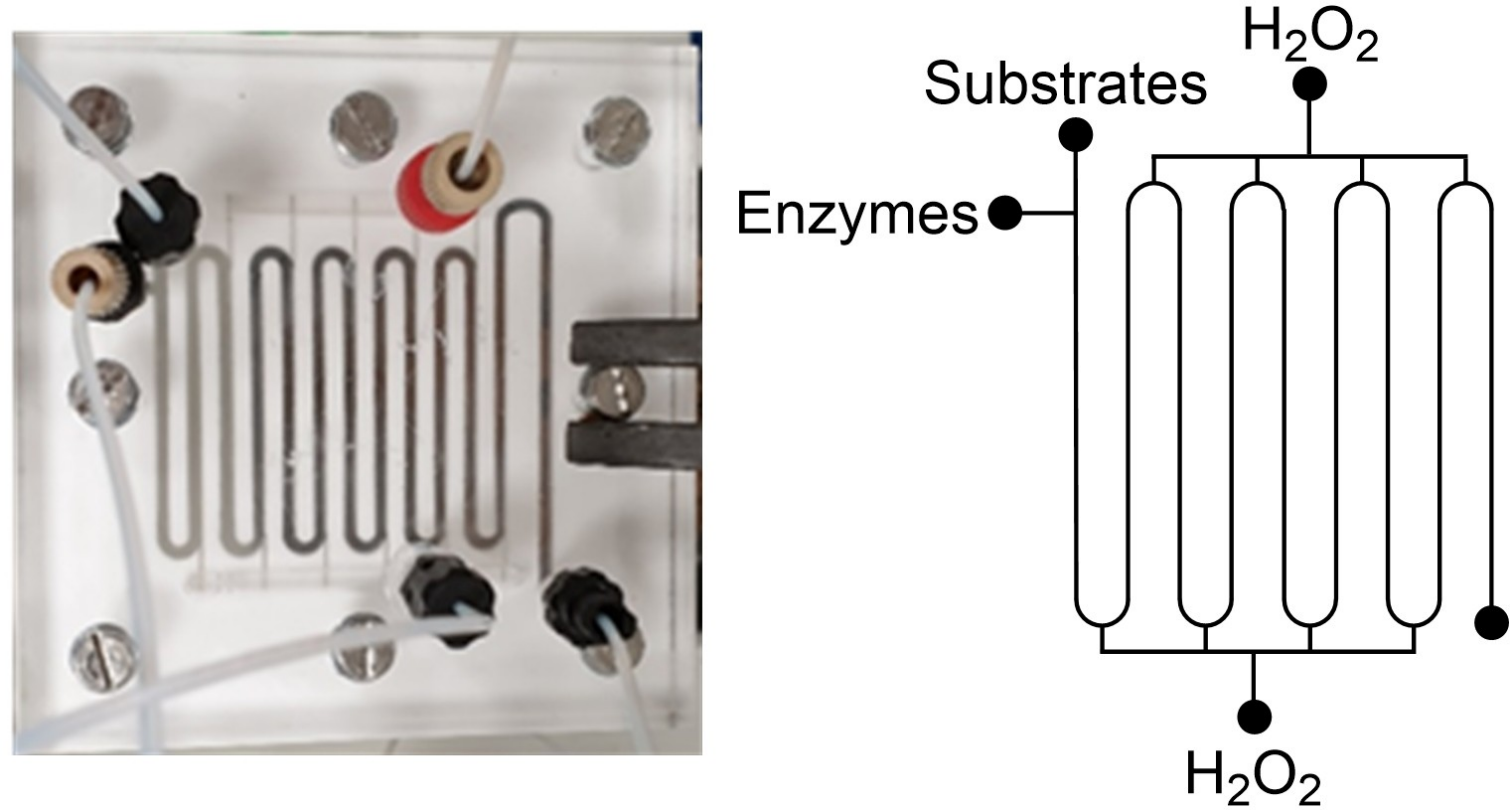
Diagrammatic representation of MPIR and picture of actual equipment

The obvious way to increase oxygen solubility is by increasing pressure (as per Henry's law, which states a linear correlation between pressure and solubility), but this leads to safety issues as already mentioned. Chapman et al. reported a new reactor design that safely produced soluble oxygen at supersaturated concentrations (Figure 1).^[27]^ A multipoint injection reactor (MPIR) was described, whereby reservoirs of hydrogen peroxide were rapidly converted to oxygen by the fast acting enzyme catalase. This allowed up to 10 times above ambient oxygen concentration to be achieved (80 mg L^−1^, ∼2.5 mM), whilst avoiding any dangerous concentrations of H_2_O_2_ which could have impacted enzyme stability.

In practice, a variant of galactose oxidase (GOase M_3–5_) performance was significantly improved and demonstrated on a panel of 15 benzyl alcohols. The residence time was as low as eight minutes at 60 mM substrate concentration (reactor volume=2.6 mL, enzyme concentration=1.9 U mL^−1^) in some cases reaching near full conversion. That represented double the substrate concentration compared to batch control experiments, that only reached around 40 % conversion (Table 1).

**Table 1:** Comparison of batch and flow GOase M_3–5_ oxidation of benzyl alcohol. S.T.Y.=Space Time Yield

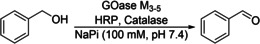		
	Batch	MPIR
Substrate conc.	30 mM	60 mM
Conversion	40 %	92 %
*t*_res_	24 h	8 mins
S.T.Y.	54 mg L^−1^ h^−1^	168 g L^−1^ h^−1^

Other enzymes shown to be improved using the MPIR include monoamine oxidase (which was used in whole‐cell form),^[27]^ other GOase variants,^[28]^ and an engineered choline oxidase.^[29]^ A primary limitation of the MPIR arises from the fact it uses soluble enzymes. The cost of recombinant enzymes can be limiting when scaling a bioprocess. Therefore, any reactor that could use the same principle of *in situ* oxygen generation but with immobilised enzymes would be attractive from a scale up perspective.

Another reactor type that has been used for improved oxygen‐supply for biocatalytic reactions is the tube‐in‐tube reactor (TiTR).^[30]^ It consists of two tubes, with the innertube made of Teflon AF‐2400, a fluoropolymer that has typical Teflon properties but is also highly gas permeable. Pressurisation of the outer‐tube allows high levels of oxygen within the innertube and hence above ambient reaction conditions to be achieved. The innertube runs along the entire reactor providing an extremely high surface‐to‐volume ratio, allowing for a much more effective supply of oxygen throughout the reaction mixture.

**Scheme 3:**
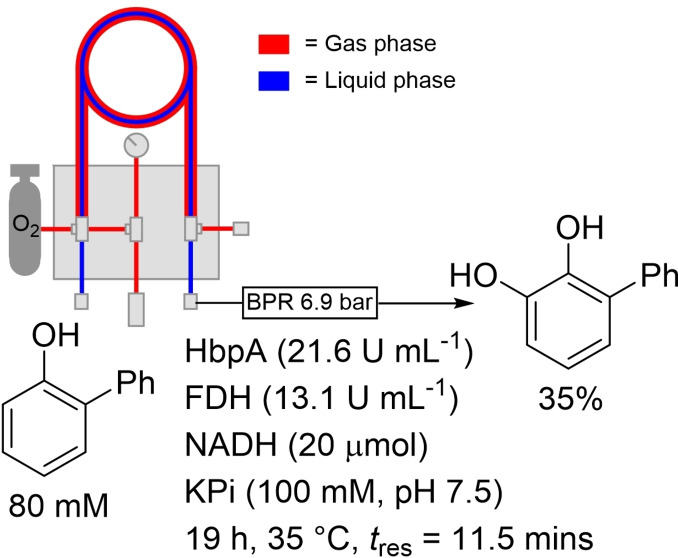
Tube‐in‐tube reactor used for hydroxylation of 2‐phenylphenol.

From a synthetic perspective, the TiTR has been used for the biocatalytic oxygenation of 2‐phenylphenol to afford 3‐phenylcatechol using 2‐hydroxybiphenyl 3‐monooxygenase (HbpA), as reported by Buehler and co‐workers (Scheme 3).^[31]^ The reactor had a total volume of eight mL and only required a *t*_res_ of 11 minutes. They reported the isolation of 740 mg of product after eight cycles, which represented an isolated yield of only 35 %. This must obviously be weighed against the speed with which it could be produced for overall efficiency. In the end, the authors calculated that 17 μmol of enzyme had been used in the preparative flow reaction, which delivered a total turnover number (TTN) of 6217 mol_product_ mol_enzyme_^−1^. The amount of enzyme used was significant though, with the authors reporting 197 U g_product_^−1^ (U=one μmol of NADH consumed in one minute at 30 °C). Clearly, metrics such as these demonstrate why reactors that operate with immobilised enzymes present as more attractive economic options for scaling of continuous biocatalysis.

Woodley and co‐workers have also demonstrated that the TiTR can be used for accurate kinetic data collection, as mentioned above.^[26]^ The application of the TiTR as a preparative scale reactor is hampered by the fragility of the Teflon AF2400 tubing. The material snaps quite easily at longer lengths, limiting its large scale application, as stated by Buehler and co‐workers.^[31]^

A simpler solution to oxygen‐limitation is to simply use a plug‐flow reactor with intermittent phases of liquid and gas, such as the recent report from Romero‐Fernandez and Paradisi.^[32]^ The authors reported a system for the synthesis of betazole from the respective alcohol using an oxidation/transamination process. To recycle the NAD^+^ used by the ADH for the oxidation, an NAD(P)H oxidase was used (NOx). The NOx mediates the oxidation of NAD^+^ to NADH at the expense of O_2_, and only produces water as a by‐product. As with previous examples, this meant that an efficient supply of oxygen was necessary to ensure enzymatic turnover. A comprehensive optimisation resulted in a system where the effluent had to be recycled four times across the immobilised enzymes to reach 66 % conversion at 50 mM substrate, with air pockets alternating with the reaction and providing O_2_. The authors calculated a transfer rate of 1.012 μmol min^−1^. This equated to a consumption of 0.39 μmol min^−1^ in their 50 mM reaction run. The biocatalyst productivity of this system was stated at 4.8 μmol_product_ mg_enzyme_^−1^, which works out as 0.53 g_product_ g_enzyme_^−1^ after five hours of operation. This would obviously have to operate for much longer to demonstrate synthetic utility, however, the simple passing of air through the reactor is a much cheaper and more straightforward way to introduce the gas to a system than some of the others presented here.

Oxygen obviously provides a massive benefit for oxidation chemistry due to the lack of significant by‐products and low cost. But the challenge of introducing it efficiently to any catalytic process has proven one of the most lasting challenges for process chemists and engineers. This is also true for biocatalysis researchers, but it can be seen that flow now has the potential to deliver solutions for oxygen supply, and improve how oxygen‐dependent biocatalysts perform in important synthetic routes.

### Hydrogen

Whilst oxygen is the most atom economic oxidant, hydrogen gas remains the cheapest reductant available. This is important for nicotinamide‐dependent biocatalysts that provide hydride equivalents during biocatalytic reduction reactions. The use of nicotinamide is costly, so the biocatalysts are coupled to recycling systems mentioned earlier including GDH (glucose oxidized to gluconolactone) and FDH (formate oxidized to CO_2_). The feedstocks are obviously cheap and readily available, but also generate significant waste as by‐products. Therefore, being able to replace these with molecular hydrogen offers a more sustainable and lower impact way to reduce nicotinamide usage.

The Vincent group have recently described several enzymatic systems that use H_2_ gas to drive biocatalytic reduction reactions. Initial reports showed sluggish reaction times, however optimisation of the flow reactor setup and identification of more active biocatalysts allowed efficient flow systems to be demonstrated.[^33^, ^34^] They reported the use of a soluble hydrogenase (SH) from *Ralstonia eutropha*, which catalysed the reduction of NAD^+^ to NADH using hydrogen gas.^[34]^ The authors stated 10 U of the SH was immobilised via adsorption to a carbon nanotube coated reactor, with a continuous production of NADH for >seven hours delivering a TTN of 6077 and a turnover frequency (TOF) of 11 min^−1^.

**Scheme 4:**
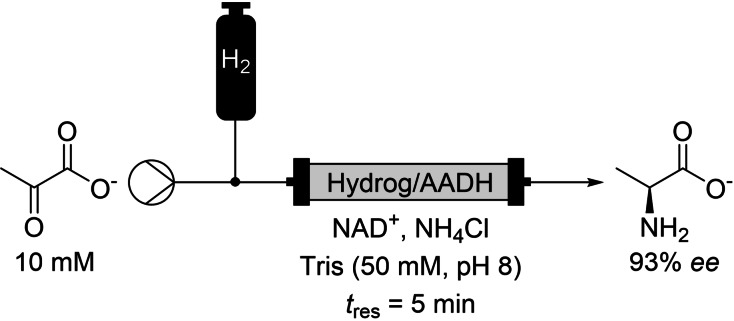
H_2_‐driven biocatalysis for the synthesis of chiral amines.

The SH was coupled to both amino acid dehydrogenase and ketoreductase to allow for continuous reductive amination and ketone reduction, all driven by the hydrogen‐gas being supplied to the reactor (Scheme 4). Impressively, they were also able to use D_2_O for the buffer which enabled H_2_‐driven biocatalytic deuteration.

This method could lay the groundwork for improvement of many H_2_‐dependent bioprocesses. For example, a host of hydrogenase‐coupled processes reported by Paul and co‐workers could be primary candidates for improvement by transfer to a continuous reactor.^[35]^ The scale of the reactor did limit the study reported by Vincent and co‐workers, with reactors no bigger than 250 μL used.

### Multistage Enzyme Reactions in Flow

The most efficient way chemists have found to apply biocatalysts has been in biocatalytic cascade reactions.[^36^, ^37^] Due to the exquisite chemoselectivity of many enzymes, it is possible to have several in the same reaction with little cross‐reactivity. Nevertheless, sometimes the reaction requirements for different biocatalysts can leave combination in the same vessel challenging. An example is metal dependent‐biocatalysts that can be deactivated by coordinating reagents, such as amines. Additionally, tandem enzymes which operate in a reversible fashion can lead to equilibrium issues depending on the thermodynamic stability of the products and the starting materials. Furthermore, efficient combination with chemocatalysis represents a major hurdle in taking full advantage of biocatalysis in synthesis, and is extremely challenging in batch processes. This section will discuss how flow has enabled the development of new cascade reactions involving biocatalysts, that would not be achievable under standard batch conditions.

#### Continuous chemoenzymatic reactions

The challenge of developing cascade chemoenzymatic processes is much greater than purely biocatalytic versions. This is due to compatibility issues including low solubility in complementary media, different requirements such as temperature or the need for an inert atmosphere, and sometimes a poor match of kinetics. Compartmentalisation can offer a route towards the combination of incompatible (bio)catalysts. Compartmentalisation in batch has been achieved, most notably by Gröger and the application of a polydimethylsiloxane (PDMS) membrane to allow *in situ* separation of sequential reactions.^[38]^

**Scheme 5:**
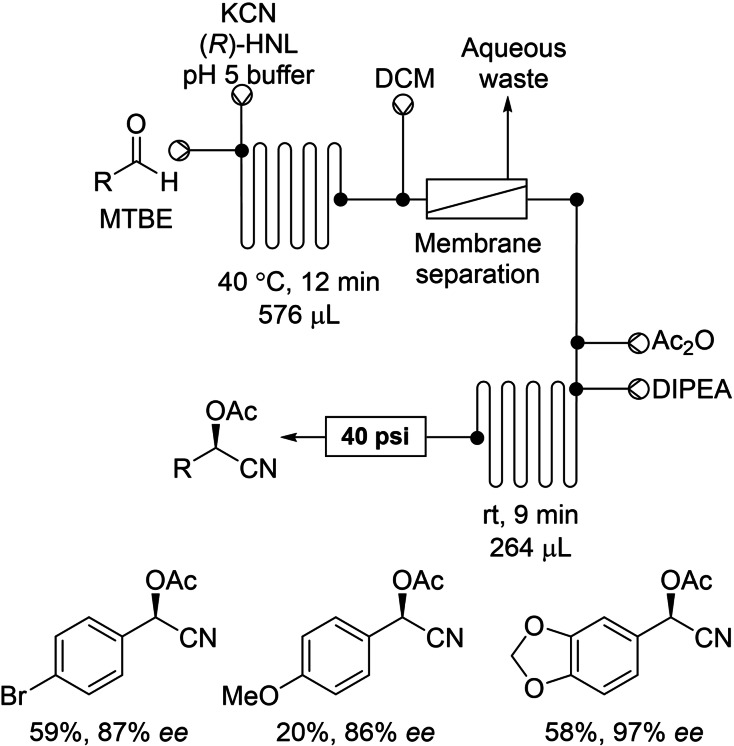
Two‐stage continuous chemoenzymatic synthesis of protected cyanohydrins.

The use of flow has also been used to enable several examples of chemoenzymatic reactions that would otherwise have not worked.^[39]^ One area flow chemistry has delivered benefits is the handling of dangerous reagents.^[40]^ The smaller reactor sizes can allow lower amounts of dangerous or toxic reagents to be used, or even generated *in situ*. Many of these reagents are low molecular weight so offer high atom economy if they can be used. The Rutjes group managed to exploit microreactors to generate HCN *in situ* for the hydroxynitrile lyase (HNL) mediated synthesis of cyanohydrins.^[41]^ Cyanohydrins are useful intermediates for synthesis, however chiral cyanohydrins tend to racemise so the authors reported the use of liquid‐liquid extraction to combine the enzymatic transformation with an inline acetylation of the free alcohol to afford the protected cyanohydrins (Scheme 5).

The exact amount of (*R*)‐HNL used was unclear, with the authors reporting a 10 % v/v solution of lysate and 0.23 M solution of aldehyde. The ratio of enzyme:substrate solution in the reactor was 5 : 1, and the conversion was reported at 88 %. Without an enzyme concentration it is not possible to determine the absolute productivity, but a six‐fold dilution of the aldehyde solution would mean a final concentration in the reactor of around 38 mM. At 88 % conversion, with a 576 μL reactor and 12 minute reaction time this would mean roughly 20.4 mg h^−1^ production of the 2‐bromobenzaldhyde derivative, which is a reasonable productivity for a 576 μL reactor. This of course would have to be compared with enzyme productivity to make a full assessment of the efficiency of the biocatalytic transformation.

**Scheme 6:**
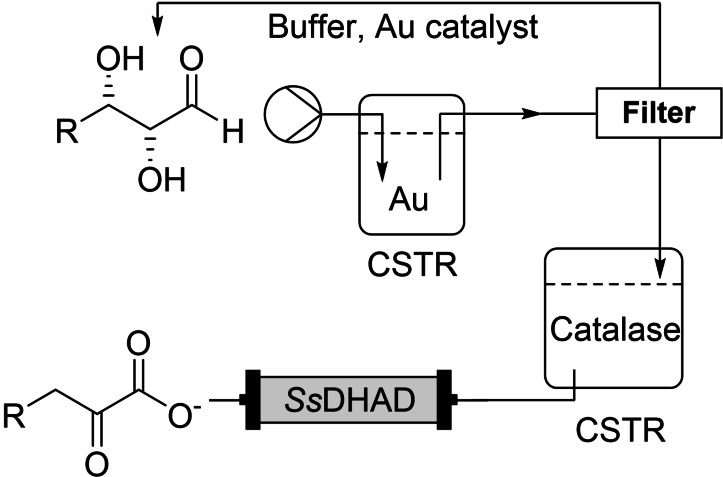
Chemoenzymatic conversion of carbohydrates to 3‐deoxy‐2‐keto sugar acids. CSTR=continuous stirred tank reactor.

In a biomass upcycling‐based approach, Sieber and co‐workers demonstrated the combination of a gold catalyst with the dihydroxyacid dehydratase from *Sulfolobus solfataricus* (*Ss*DHAD) to afford carbohydrate‐derived 3‐deoxy‐2‐keto sugar acids (termed KDS).^[42]^ In batch the reaction did not proceed due to deactivation of the Au catalyst upon addition of the enzyme, through presumed coordination of the iron‐sulphur cluster of the enzyme to the Au catalytic centre. A hollow fibre membrane was used to separate the reactions, with a ‘catalase’ reactor in between to remove the H_2_O_2_ by‐product of the Au‐oxidation (Scheme 6).

The flexibility and efficiency of the system was demonstrated by production of the KDS derivatives of d‐glucose, d‐galactose, l‐arabinose, and d‐xylose, all at average conversions of at least 31 mM and as high as 41 mM. It seemed the column that was used had a 1 mL volume, although there was little additional detail given with respect to enzyme loading in the immobilised column, preventing a calculation of enzyme productivity. The authors stated a 58 % yield of the l‐arabinose KDS derivative from a 100 mL reaction volume after purification.

The system clearly showed its flexibility, and necessity, over the batch equivalent, however the final set up was relatively complex to engineer. It highlights the difficulty of combining chemo‐ and biocatalysis, whilst also demonstrating the benefits that can be derived from it. Chemoenzymatic reactions offer a much greater access to new chemical entities than application of the individual disciplines. The problem, however, is the combination of the often opposing conditions that are required to enable efficient reactivity. As stated, there have been multiple solutions proposed in batch, including simply mixing the catalysts together,[^43^, ^44^] several examples of membrane separation,[^38^, ^45^] and recently *in situ* compartmentalisation through surfactant use.[^46^, ^47^] Whilst these examples demonstrate the possibility of batch chemoenzymatic reactions being possible, the engineering of these reactions provides many additional processing problems that must be considered, especially when considering scale up. Here is where flow can provide simpler solutions: compartmentalisation is more straightforward, and in the context of packed‐bed reactors immobilised (bio)catalysts can be allowed to operate simultaneously under separate, optimum reaction conditions. The technology that is used anyway for flow (columns, filters) are in place to enable separation of different types of reactions, and do not have to be considered as an extra point as in batch (membrane choice, surfactant usage, etc.). This should reduce optimisation time when implementing continuous chemoenzymatic reactions versus their batch equivalents. Ultimately, it should provide a platform to discover and develop novel chemoenzymatic synthetic sequences in a more efficient manner.

#### Continuous biocatalytic cascades

Despite the famed orthogonality of many biocatalytic reactions, leading to the development of one‐pot cascade systems,^[36]^ there are instances where multi‐step biocatalytic reactions could benefit from compartmentalisation.^[48]^ Take for example a recent report from scientists at GlaxoSmithKline, where a reductive aminase (RedAm) was evolved for the manufacture of a phase II clinical candidate for leukaemia.^[49]^ To increase efficiency, the authors attempted to combine the RedAm with an alcohol dehydrogenase to produce the aldehyde substrate for the reaction in a closed‐loop borrowing hydrogen cascade.^[50]^ However, the thermodynamics of the one‐pot process meant that only about 50 % conversion to the amine could be achieved, and they reverted to the Cu‐mediated oxidation of the starting alcohol in a step‐wise process instead. Had this been transferred to flow, continuous removal of the amine product could have potentially helped shift the equilibrium towards the product.

**Scheme 7:**
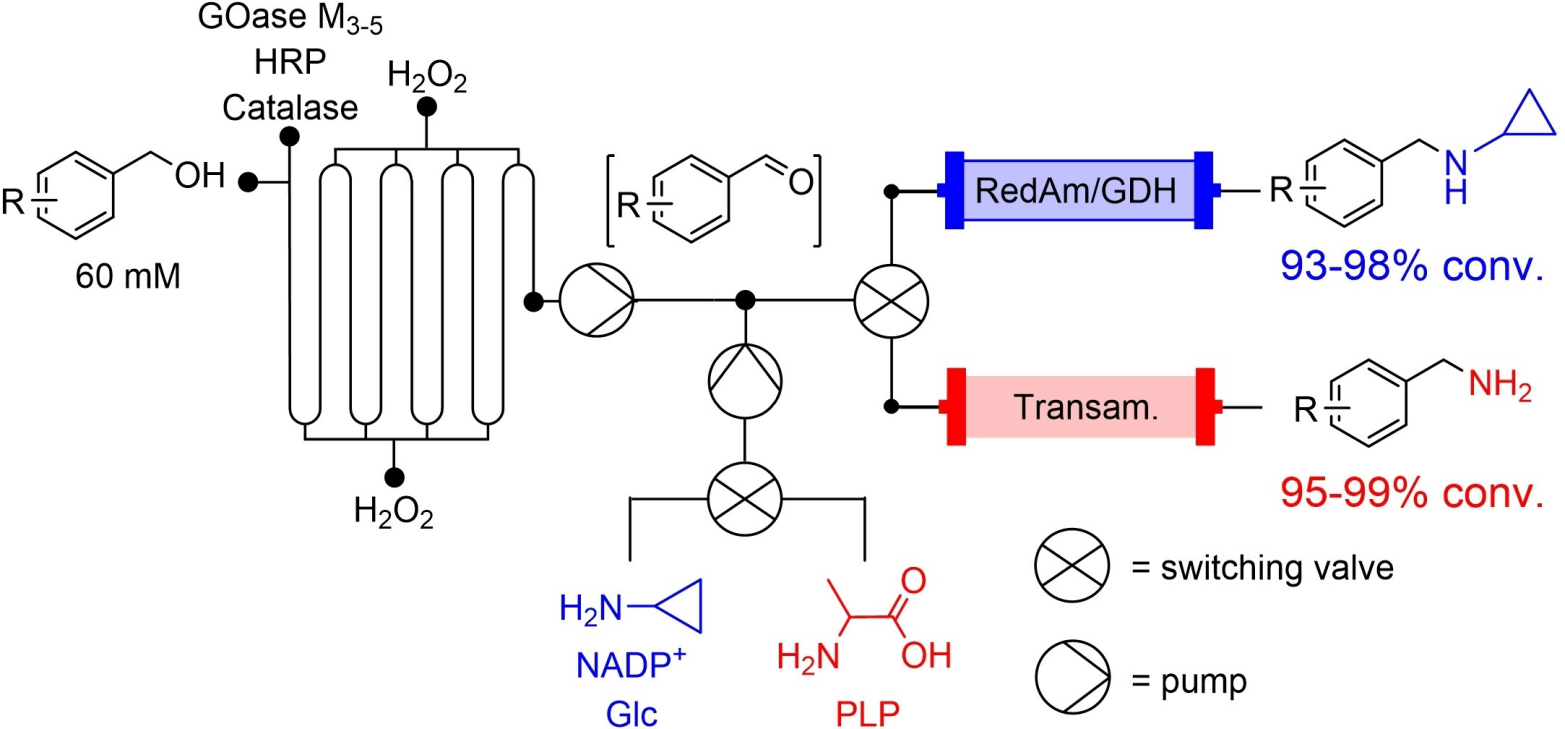
Multistage continuous biocatalytic cascades not attainable in batch.

Another instance is the combination of enzymes that are incompatible. The example given earlier, whereby metal‐dependent enzymes may not work in the presence of coordinating amines, was demonstrated to work when transferred to a continuous process recently.^[29]^ Mattey et al. reported a combination of different reactors to enable biocatalytic cascades that were not possible under standard batch conditions. First they applied the MPIR (Figure 1) to improve an engineered choline oxidase,^[51]^ producing aldehydes which were then flowed through columns containing different aminating enzymes (RedAm for secondary amines and transaminase for primary amines). The choline oxidase/RedAm cascade mirrored an earlier report from Ramsden et al.^[52]^ The batch reaction for the synthesis of *N*‐allylcinnamylamine proceeded with a space time yield (STY) of 0.13 g L^−1^ h^−1^, and productivity of 0.13 g h^−1^ g_IRED_^−1^. In flow, the same cascade delivered a STY of 2.1 g L^−1^ h^−1^, and a biocatalyst productivity of 0.14 g h^−1^ g_IRED_^−1^. This would of course be improved by leaving the reactor to go for longer (the flow reactor was only run for four hours), with other work demonstrating that IREDs can be left for days without losing activity.^[53]^ The GOase M_3‐5_ variant was also combined with aminating enzymes to afford a range of primary and secondary benzylamines. This reaction was shown not to work under typical batch conditions, underlining the necessity of using this flow system for this biocatalytic cascade (Scheme 7).

This work has demonstrated that enzymes that are not compatible can be made to work together using flow systems. The limitation of the system described above is the lack of automation, with all switching between columns and reactors performed manually. In addition, no form of in‐line analysis was incorporated, with offline gas chromatography or mass spectrometry used.

By moving towards automated flow systems, the efficiency could be increased significantly, and offers further opportunity to access process improvements in biocatalytic reactions that are still yet to be reached.

### The Future of Flow Biocatalysis: Data‐Driven Improvement and Automation

The ever‐increasing demand for higher throughput in synthetic chemistry poses a major challenge to industry. The rate at which new compounds can be tested and brought to market is largely dependent on the speed and scalability of their syntheses. Automated synthesis is perfectly poised to challenge traditional synthetic processes whilst facilitating a new wave of innovation by increasing access to (un)known compounds.^[54]^ The versatility and flexibility of flow chemistry means it is now seen as a technology that can be the perfect vehicle to enable automated synthesis.^[55]^ The following section will highlight some of the recent developments in the general area of flow, and pose how this may further enhance the field of flow biocatalysis. Furthermore, computer‐aided planning programmes, which will have a significant part to play in fully automated systems, are discussed.

#### Automated multistep synthesis in flow

Most chemical compounds are synthesized in multiple steps and often carried out using a telescoped approach where intermediate separation is not required. This approach is ideal for implementation in continuous mode where multiple technologies can be used to synthesize complex molecules. The integration of automation into these multi step reactions in flow allows chemists to quickly generate a large number of compounds with minimal physical intervention.^[56]^

A number of automated flow platforms have been developed for the multi‐step synthesis of peptides, oligonucleotides and polysaccharides.[^57^, ^58^] Previously, automated synthesis was restricted to polymeric syntheses; however, Collins et al. recently reported a general multi‐step synthesizer for small molecules that was capable of generating 87 % of computable FDA approved small molecule drugs.^[54]^

The diversity of chemical reactions means a general automated synthesizer must be able to perform multiple reactions and different types of operations (e. g. catalysis, purification etc.) whilst being able to facilitate efficient telescoped reactions.^[55]^ Perhaps the most common approach to this is to adopt a modular system that can allow for different reaction conditions to be achieved in individual modules (i. e. different temperatures, pressures etc.). Bédard et al. showed the versatility of such a modular system which consisted of multiple reactor types (packed bed, photochemical), an inline extraction unit and inline analytics via HPLC, FTIR and MS. The entire system was also computationally controlled on a closed loop system which allowed for self‐optimization.^[59]^ This system was further enhanced with the integration of machine learning based computer‐aided synthesis planning (CASP) and enabled autonomous recommendations of reaction pathways based on a target molecule. The automated flow platform was used to synthesize 15 medicinally‐relevant compounds continuously, only requiring minimal input to ensure reaction compatibility with the flow set up.^[60]^

This modular approach used in chemical synthesizers could also be used in biocatalytic reactions in flow. It has been shown that batch incompatible cascades can be achieved using modular flow systems (Scheme 6) and can be used in a ‘closed loop’ for self‐optimization.[^19^, ^29^, ^53^] The use of highly stabilised immobilized enzymes in fixed‐bed reactors can facilitate a versatile system that allows multiple reaction types to be carried out sequentially. Additionally using this standard laboratory equipment allows for easy integration into other flow components allowing for inline purification and analysis as exemplified by Paradisi and colleagues.^[61]^

#### Inline analytics and continuous monitoring

The continuous analysis of multistep synthesis allows for enhancement of reaction control via closed loops systems. The integration of process analytical technology (PAT) into continuous flow systems enables real‐time reaction monitoring and can be applied to several methodologies such as reaction kinetic analysis, process control and self‐optimization.^[62]^ In a striking example the Kappe group integrated NMR, UV/Vis, IR and UHPLC into a multistep reaction platform. The system was used for the synthesis of the API mesalazine with a productivity of 1.6 g h^−1^.^[62]^ Continuous monitoring of biocatalytic reactions has also been studied. Röther and co‐workers continuously monitored a carboligase‐transaminase cascade via a benchtop NMR connected to a CSTR. This approach allowed for full detection of all substrates and products in aqueous buffer and did not require the addition of any deuterated solvents.^[63]^ This isolated example demonstrates that it is possible to incorporate analytics directly into continuous flow biocatalysis. This obviously allows for exciting developments aligned to those discussed above, such as product monitoring or self‐optimisation. It could also present an opportunity for advanced real‐time kinetic analysis of enzymatic transformations to take place (e. g., flowNMR for intermediates and active site species), offering a new era in the collection of active site reaction data for biocatalysis.

#### Computer aided route planning

Though many automated synthesis platforms reduce physical intervention, the vast majority still require time consuming tasks to be performed manually. A major barrier to adopting a fully automated system is the initial design of synthetic pathways. The availability of most chemical reactions reported in history via programs such as Reaxys^TM^ or SciFinder^TM^ has made it possible to construct syntheses purely based on data as opposed to physical experimentation.^[64]^ The ever increasing availability of data also allows machine learning approaches to be used to further enhance the success rate of proposed routes.^[65]^

#### Computer aided retrosynthesis

Retrosynthesis was one of the most important advances in organic synthesis when first formalized in the 20^th^ Century by Corey. The skill of retrosynthesis, however, is generally considered to be developed over the course of a career, gradually building knowledge of the possible disconnections that can be made. To address this issue, the power of computation has recently come to the fore to allow augmentation of retrosynthesis, leading to several CASP programmes to be created.^[66]^ The example summarized previously by the Jamison and Jensen groups illustrates how continuous processing can seamlessly incorporate CASP and machine learning to enhance synthetic planning and increase route efficiency.^[60]^

A common omission from these CASP programmes is biocatalysis. Without the incorporation of enzymatic disconnections, there is less chance of people discovering new biocatalytic reactions that could enhance their syntheses.^[67]^ Finnigan *et al*. have gone some way towards addressing this imbalance. They recently reported an open access online tool called RetroBioCat, a CASP that could be used in the planning of biocatalytic cascades.^[68]^ Here RetroBioCat was used to automate critical steps in assessing the feasibility of a biocatalytic pathway whilst only requiring the target molecule as an input.[^68^, ^69^] This combination of data science and biocatalysis should allow for seamless integration into automated continuous flow systems.

#### Automated pipeline for biocatalysis

Machine learning approaches allow the repurposing of a biocatalyst in a more automated approach, and can drastically improve the development speed of a biocatalyst. Recently a huge leap in computer aided biocatalyst design was disclosed by Jumper et al. Here the first computational method for predicting protein structure was proposed via a neural network‐based model (AlphaFold). This system allowed the solving of complex modelling problems whilst also providing insights into the function of yet unknown proteins.[^70^, ^71^] This AI assisted approach will potentially allow for the generation of ‘designer’ biocatalysts with very minimal experimentation required.

**Figure 2:**
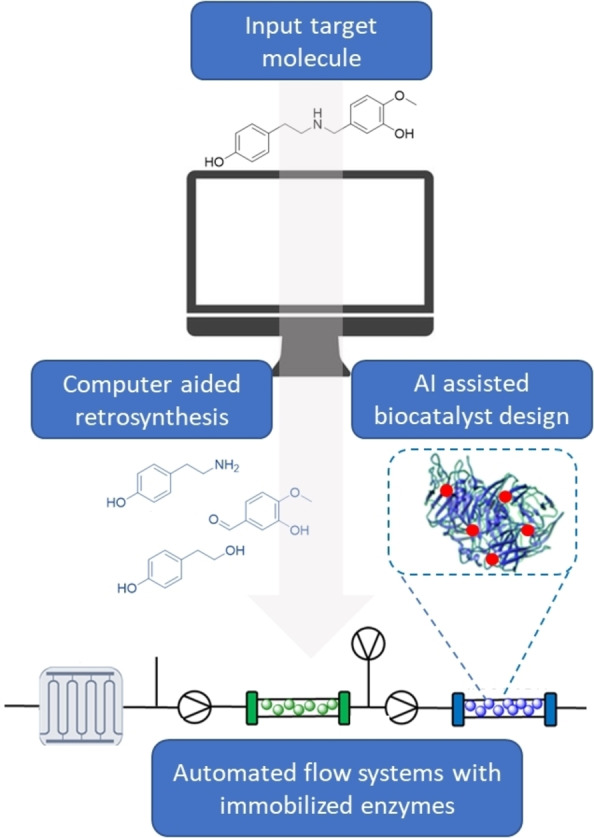
Computer aided workflow for adopting fully autonomous biocatalytic reactions in continuous flow.

Moving forward, the integration of automation into all developmental stages of a biocatalytic process (Synthesis route planning, biocatalyst design and continuous reactions) will not only improve throughput but also de‐risk the option of adopting an enzymatic reaction step (Figure 2). Continuous reactors are central to the success of this adoption.

### Outlook for Continuous Flow Biocatalysis

It can be envisaged that increasingly more complex biocatalytic cascades will continue to be discovered and designed, akin to the recent islatravir example from the Merck process laboratory.^[72]^ The challenge for researchers will be to apply retrosynthetic tools to identify new disconnections that appear at first unconventional, then for others to also consider how technology could facilitate the forward reaction. Using computational tools can assist route design, while continuous reactors can provide a viable option for reactions that may not be feasible under standard conditions. Furthermore, to enable uptake in early‐stage discovery, research should focus on how flow can increase the pace of biocatalytic transformations. For example, the microfluidic platform described by Holland‐Moritz for continuous screening of whole‐cell engineered enzyme variants on nL scale was able to screen at a rate of 0.7 samples s^−1^.^[73]^ Adaptation of a systems such as this will allow incorporation of multiple analytical methodologies, and could facilitate the move away from plate‐based screening methods which are dependent on colourimetric assays. This could significantly increase the rate of discovery of new biocatalytic transformations.

Flow biocatalysis is beginning to reach a level of maturity that will potentially see it become a mainstay in the chemist's toolbox. Advancements such as those that allow reactions that cannot be performed in batch to work, coupled to the significant automation developments that have been made in recent years, offer the chance to push the capabilities of enzymes well beyond their current limits.

## Conflict of interest

The authors declare no conflict of interest.

1

## Biographical Information

***Sebastian Cosgrove** obtained an MChem degree from the University of Manchester, then moved to the University of Leeds where he completed his PhD in synthetic organic photochemistry with Prof Steve Marsden. He then moved to the Manchester Institute of Biotechnology to work with Profs Nick Turner and Sabine Flitsch as a PDRA for several years, before joining the Future Biomanufacturing Research Hub with Prof Nigel Scrutton as a Research Fellow in continuous flow biocatalysis. In November 2020, he started his independent career at Keele University. His research interests include biocatalysis, continuous flow and enzyme immobilisation*.

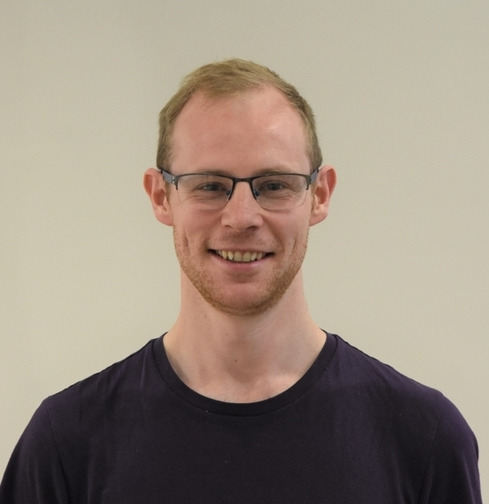

## Biographical Information

***Ashley Mattey** obtained his Ph.D. at the University of Manchester under the supervision of Prof. Sabine Flitsch and Prof. Nick Turner working on the use of oxidases in enzymatic cascades both in batch and flow. He is currently a researcher at Enginzyme AB working on enzyme immobilization and flow biocatalysis. He is interested in the integration of automation in continuous biocatalytic processes*.

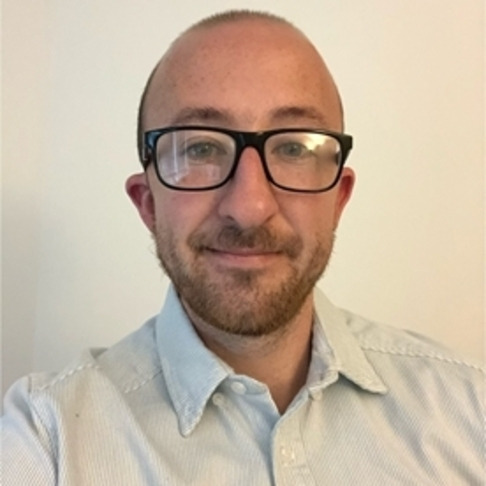

## Data Availability

Data sharing is not applicable to this article as no new data were created or analyzed in this study.
